# Dr. Cleverly

**Published:** 1825-01-01

**Authors:** 


### OBITUARY.

### DR. SAMUEL CLEVERLY.

Among the numerous cases of distress to which the attention of benevolent persons has been solicited, few have occurred more deplorable than that occasioned by the death of Dr. Cleverly, who was cut off in the prime of life, leaving a Wife and five Sons (the eldest fourteen) utterly in a destitute state.

This Gentleman fell a victim to a fever of only five days duration, which attacked him while engaged in his professional duties.

His early prospects were blighted by his detention in France for eleven years, during which his kindness to his fellow prisoners, both professionally and in a pecuniary point of view, was unremitting.

Dr. Cleverly, who was Physician to the Orphan Asylum as well as the Fever Hospital; was a Licentiate of the College of Physicians, and highly esteemed by his professional brethren.

After this appeal is made to the feelings of Medical Men, the friends of the family trust that the Public at large will be interested in the case of Dr. Cleverly, who died in their service.

The following Gentlemen have formed themselves into a Committee to promote a Subscription, and will receive the same :—

Dr. Babington, Aldermanbury
Dr. Merriman, 34, Brook Street
Dr. W. Philip, Cavendish Square
Dr. Sims, 37, Wimpole Street
Dr. Cooke, Gower Street
Dr. Darling, 6, Russel Square
Dr. Clutterbuck, Bridge-st. Blackfriars
Dr. Bright, 14, Bloomsbury Square
Dr. Billing, 5, Bedford Place
Dr. Gordon, 2, Finsbury Square
Dr. Conquest, 45, Finsbury Square
Dr. Scudamore, 6, Wimpole Street
Dr. Armstrong, Russell Square
Dr. James Johnson, Suffolk Place, Pall Mall East
Dr. Henderson, 6, Curzon Street
Dr. Warburton, 5, Clifford Street
Dr. John Sims, 29, Cavendish Square
Dr. Granville, Grafton Street, Berkeley Square
Dr. Outram, Hanover Square
Dr. Robinson, New Broad Street
Dr. Ayre
Dr. Birkbeck, New Broad Street
Dr. Tweedie, Ely Place
Dr. Filkin, 48, Great Coram Street, Russell Square
Dr. Temple, Bedford Row

Sir A. Cooper, Bt. Spring Gardens

J. Abernethy, Esq. 14, Bedford Row

B. Travers, Esq. Broad Street

H. Cline, Esq. Lincoln's-Inn-Fields

E. Stanley, Esq. Lincoln's-Inn-Fields

H. Earle, Esq. George-st. Hanover-sq.

B. C. Brodie, Esq. Saville Row

F. Copeland, Esq. 4, Golden Square

H. Alexander, Esq. 6, Cork Street

J Stevenson, Esq. 18, Margaret-st.

J. Wardrop, Esq. Charles Street, St. James's Square

W. Maule, Esq. 185, Piccadilly

M. Ware, Esq. 22, Bloomsbury-sq.

R. R. Pennington, Esq. 21, Montague Place

R. Verity, Esq. Bolton-st. Piccadilly

W. Acret, Esq. 1, Francis Street, Bedford Square

Jos. Hayes, Esq. Upper Charlotte-st, Fitzroy Square

D. Price, Esq. 9, Upper East Smithfield

Samuel Plumbe, Esq. Great Russell Street, Bloomsbury

J. Symes, Esq. 80, Judd Street

H. D. Jones, Esq. 7, Gt Newport-st.

J. Morgan, Esq. Bedford Row

Dr. Uwins, 13, Bedford Row

Dr. Richardson, 37, Bedford Square

Dr. Burrowes, Gower Street

S. Cartwright, Esq. 32, Old Burlington Street

Dr. Shepherd, 14, Russell Square

Charles Munay, Esq. John Street

A. Barker, Esq. Northern Dispensary, New Road

Dr. Roget, 39, Bernard Street, Russell Square

Dr. Bostock, 22, Upper Bedford Place

W. Drew, Esq. Gower Street

E. Forster, Esq. of the Banking House of Sir John Lubbock, Bt. & Co. 11, Mansion House Street

J. L. Mallet, Esq. 52, Upper Gower Street, Bedford Square

Godfrey Sykes, Esq. Powis Place

John S. Taylor, Esq. Great James Street, Bedford Row

David Wallace, Esq. 1, Fludyer-st,

And at the Banking Houses of Sir John Lubbock & Co. 11, Mansion House Street; Messrs. Hoare, Fleet Street; and Messrs. Goslings and Sharpe, 19, Fleet Street; Sir Claude Scott, Bart. & Co. Hollis Street, Cavendish Square.

The following Gentlemen have kindly consented to collect any Subscriptions not paid at the Bankers:—Dr. Ayre, Dr. Filkin, Mr. Jones.

